# Bidirectional association between rheumatoid arthritis and chronic obstructive pulmonary disease: a systematic review and meta-analysis

**DOI:** 10.3389/fimmu.2024.1494003

**Published:** 2024-12-02

**Authors:** Meijiao Wang, Hejing Pan, Yingqi Zhai, Haichang Li, Lin Huang, Zhijun Xie, Chengping Wen, Xuanlin Li

**Affiliations:** The Research Institute of Chinese Medicine Clinical Foundation and Immunology, School of Basic Medicine Sciences, Zhejiang Chinese Medical University, Hangzhou, China

**Keywords:** rheumatoid arthritis, chronic obstructive pulmonary disease, bidirectional association, COPD, RA

## Abstract

**Background:**

Rheumatoid arthritis (RA) and chronic obstructive pulmonary disease (COPD) are prevalent and incapacitating conditions, sharing common pathogenic pathways such as tobacco use and pulmonary inflammation. The influence of respiratory conditions including COPD on RA has been observed, meanwhile RA may constituting one of the risk factors for COPD. It unclear that whether a bidirectional associate between RA and COPD. Our study aims to explore the bidirectional relationship between RA and COPD.

**Methods:**

We systematically searched PubMed, Cochrane Library, and Embase for observational studies from the databases inception to February 20, 2024, utilizing medical subject headings (MeSH) and keywords. We included studies in which RA and COPD were studied as either exposure or outcome variables. Statistical analyses were conducted employing STATA software (version 14.0). The relationship was reported as odds ratios (OR) and corresponding 95% confidence intervals (CI). Publication bias was assessed using funnel plots and Egger’s regression.

**Results:**

Nineteen studies with 1,549,181 participants were included. Risk of bias varied from low to moderate, with evidence levels rated as low or very low. Pooled analysis revealed a significant association between RA and increased COPD risk (OR=1.41, 95%CI 1.13 to 1.76, I^2^ = 97.8%, *P*=0.003). Subgroup analyses showed similar COPD risk elevations in both of genders, seropositive/seronegative RA, cohort and case control studies. Additionally, there was a significant RA risk increase among those with COPD (OR=1.36, 95%CI 1.05 to 1.76, I^2^ = 55.0%, *P*=0.022), particularly among females and seropositive RA, and cohort studies.

**Conclusion:**

The meta-analysis identifies a significant bidirectional association between RA and COPD, emphasizing mutually increased risk. Recognizing this connection may can inform proactive approaches to disease prevention and management, potentially reducing the public health burden and improving quality of life.

**Systematic Review Registration:**

https://www.crd.york.ac.uk/PROSPERO/, identifier CRD42024518323.

## 1 Introduction

Rheumatoid arthritis (RA) is a systemic inflammatory disease primarily affecting joints, with an estimated prevalence ranging from 0.5% to 1% of the population (1). Its societal impact encompasses substantial costs, disability, and diminished productivity. Despite advancements in diagnosis, understanding of its pathogenesis, and therapeutic options, RA remains incurable. Thus, early identification of associated risk factors could potentially delay its onset. Previous studies have explored various risk factors including genetic predisposition, familial history, female gender, exposure to tobacco smoke and air pollutants, antibiotic use, obesity, periodontitis, and atopic dermatitis (2–8). Notably, respiratory conditions such as asthma, pneumonia, interstitial lung disease, and chronic obstructive pulmonary disease (COPD) have also been implicated as influencing factors on RA (9–12).

COPD is a major cause of global mortality and disability, characterized by persistent and often progressive airflow obstruction. Individuals with RA have increased mortality associated with COPD, while RA itself serves as a risk factor for developing COPD. This association is evident due to shared risk factors such as cigarette smoking and systemic inflammation. Smoking triggers the production of anti-citrullinated protein antibodies (ACPA), elevating the risk of both RA and COPD (13, 14). Although there were studies on the relationship between COPD and RA, they may share a common pathogenesis, however, there was lack of systematic review on the bidirectional correlation between them. Consequently, we hypothesize a bidirectional association between RA and COPD, and aim to systematically review existing evidence to ascertain whether they are mutual risk factors for each other.

## 2 Methods

### 2.1 Data sources and selection criteria

We systematically retrieved articles from the PubMed, Cochrane Library, and Embase for studies published from the databases inception through February 20, 2024, with no language restrictions. The search strategy utilized Medical Subject Headings (MeSH) and keywords, including: “arthritis, rheumatoid”, “rheumatoid arthritis”, “RA”, “pulmonary disease, chronic obstructive”, “COPD”, and their variants. Details of the search strategy were shown in the **Supplementary Tables 1**-**3**. Additionally, we manually screened the reference lists of relevant systematic reviews to supplement our search and ensure comprehensive study identification (15, 16).

The observational studies were included based on the following criteria: (1) cohort studies, case-control studies, or cross-sectional studies; (2) investigating the association between RA and the risk of COPD, or the association between COPD and the risk of RA; (3) report relative risk (RR), hazard ratio (HR), or odds ratio (OR) with corresponding 95% confidence interval (CI). The studies were excluded according to exclusion criteria: (1) not the observational studies; (2) did not investigate the association between RA and the risk of COPD, or the association between COPD and the risk of RA; (3) studies lack relevant outcomes, such as did not report relative risk (RR), hazard ratio (HR), or odds ratio (OR) with corresponding 95% confidence interval (CI); (4) the studies was conference abstracts, study protocols, or letters.

### 2.2 Study selection and data extraction

Two independent reviewers (MJ Wang and HJ Pan) screened titles and abstracts, followed by full-text review of potentially eligible articles. Discrepancies were resolved through discussion with a third arbiter (XL Li). Data extraction was independently conducted by the aforementioned reviewers, following the guidelines on data extraction for systematic reviews and meta-analysis (17). We used predesigned forms for extracting data, including the first author, year of publication, study region, study period, study type, sample size, female/male, age, and diagnosis of RA or COPD. Disagreements were also resolved through discussion with the third reviewer.

### 2.3 Risk of bias

The Newcastle-Ottawa Scale (NOS) was utilized to assess cohort and case-control study quality based on selection, comparability, and exposure (18). Each study could earn up to nine stars, with four for participant selection and exposure measurement, two for comparability, and three for outcome assessment and follow-up adequacy. Higher star counts reflect higher study quality (0-3: low, 4-6: moderate, 7-9: high, respectively). The Agency for Healthcare Research and Quality (AHRQ) assessed cross-sectional study quality with 11 items, where “yes” scored 1 point and “no” or “unclear” scored 0. Scores of 0-3, 4-7, and 8-11 were defined as low, medium, and high quality, respectively (19).

### 2.4 Evidence certainty

The Grading of Recommendations Assessment, Development, and Evaluation (GRADE) system was used to evaluate evidence certainty, initially grading observational studies as low quality (20, 21). The quality of evidence can then be graded as high, moderate, low, or very low, based on rating is modified downward (such as study limitations, inconsistency, indirectness, imprecision, and publication bias), and rating is modified upward (large magnitude of effect, dose response, confounders likely minimize the effect) (22, 23).

### 2.5 Data analysis

For statistical analysis, adjusted odds ratios (OR) and corresponding 95% confidence intervals (CI) were used to assess the relationship between RA and COPD risk, and vice versa. Heterogeneity was evaluated using χ^2^-test and I^2^-values, with a fixed-effects model applied for *P*>0.1 and I^2^<50%, and a random-effects model for I^2^>50%. Sensitivity analysis ensured result robustness. Egger’s regression and funnel plot examination were used to assess publication bias. Subgroup analyses were conducted based on gender, serum indicators, and study design. All analyses were performed using STATA statistical software version 14.0.

The meta-analysis adhered to the guidelines of the Preferred Reporting Items for Systematic Reviews and Meta-Analyses (PRISMA) (24). Furthermore, the protocol was preregistered on the International Prospective Register of Systematic Reviews (PROSPERO) platform, with the approval number CRD42024518323.

## 3 Results

### 3.1 Literature search and study characteristics

A total of 2,455 records were retrieved through the search. Following title and abstract screening, 84 articles were deemed potentially relevant. Nineteen studies were finally included in the study (**Figure l**).

**Figure 1:**
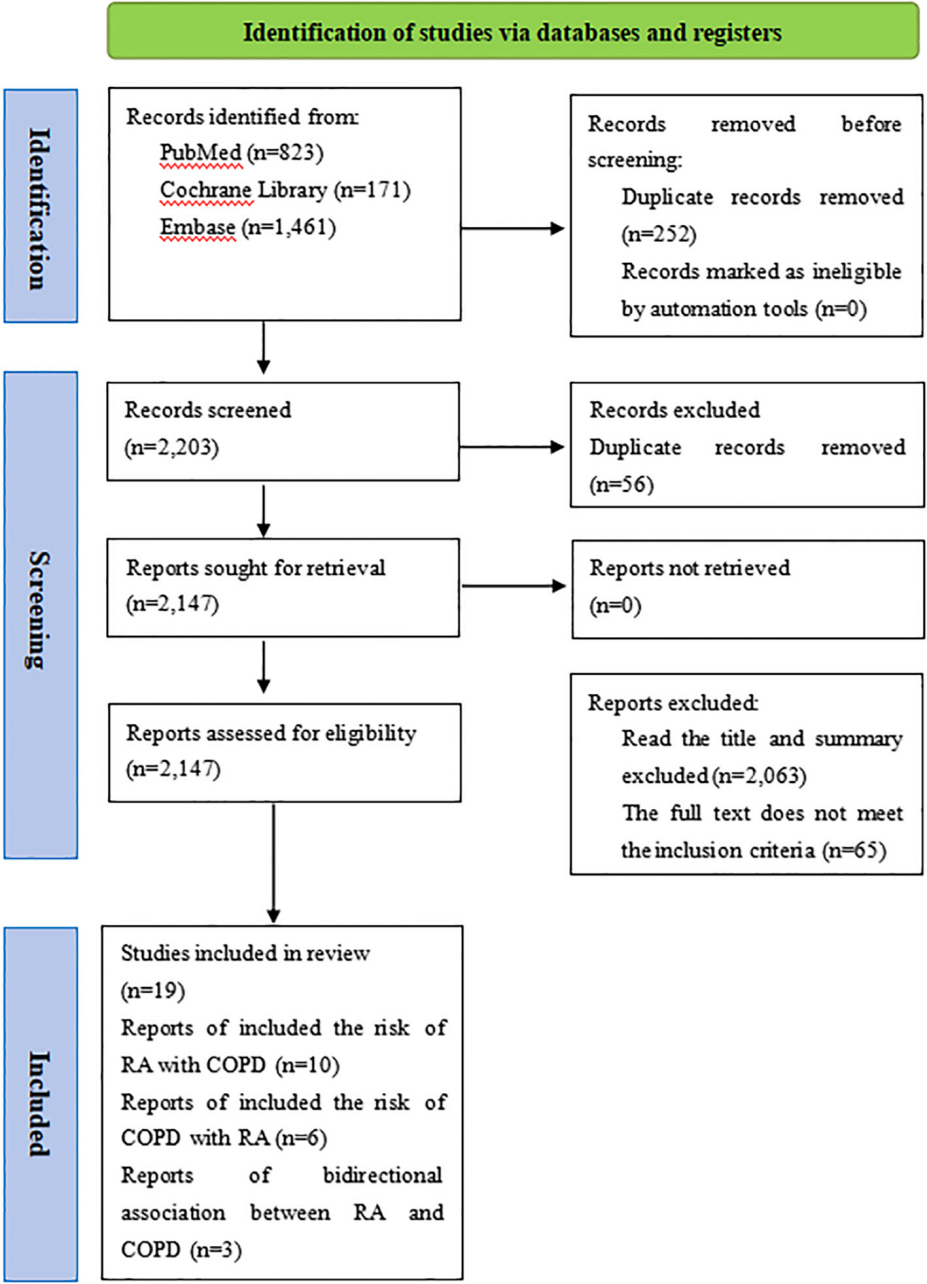
Studies screening process.

The meta-analysis included 19 studies with 1,549,181 participants, published up to February 20, 2024 (10, 25–42). Among these, 10 studies analyzed COPD as the outcome with RA as the exposure (26–28, 30, 31, 33, 35, 36, 40, 42) (**Table 1**), while 6 examined RA as the outcome with COPD as the exposure (10, 25, 29, 32, 37, 39) (**Table 1**), 3 investigated bidirectional association between RA and COPD (34, 38, 41) (**Table 1**). These studies included eight cohort, six case-control, and five cross-sectional studies, mainly from North America, Europe, and Asia. Most studies adhered to well-defined diagnostic criteria for RA/COPD, with minor discrepancies in adjusted confounders (**Supplementary Table 4**).

**Table 1:** Characteristics of the included studies.

Author	Year	Country	Study period	Study type	Study size	Female/Male	Age (years)	Diagnosis of RA/COPD
Chung C (42)	2024	Korea	January 2010 and December 2017	Cohort study	Case group: 46030, Control group: 230150	Case group: 35424/10606, Control group: 177120/53030	(57.51 ± 9.71)	RA: ICD-10
Kim JG (40)	2023	Korea	2017 to 2019	Cross-sectional study	Case group: 334, Control group: 13384	Case group: 269/65, Control group: 7522/5862	Case group: (65.7 ± 11.0) Control group: (59.5 ± 11.95)	COPD: GOLD guidelines
Yang K (41)	2023	The UK	As of March 2023	Cohort study	Case group: 4755, Control group: 391525	Case group: 3171/1584, Control group: 204299/187226	< 50, 50-59 and ≥60	RA and COPD: ICD-10
Kronzer VL (39)	2022	US	2010 to June 2020	Case-control study	Case group: 741, Control group: 2223	Case group: 563/178, Control group: 1689/534	Case group: 55 (45,63) Control group: 56 (47,63)	RA: The 2010 ACR/EULAR criteria
Jung JH (36)	2021	Korea	2008 to 2016	Cross-sectional study	Case group: 318, Control group: 27977	Case group: 260/58, Control group: 15618/12359	Female Case group: (62.2 ± 11.26) Control group: (54.9 ± 12.43) Male Case group: (61.4 ± 10.29) Control group: (55.6 ± 12.82)	COPD: (FEV1/FVC) <0.7, chronic cough or sputum for more than 3 months, and/or smoking history of ≥10 pack-years
Kronzer VL (37)	2021	Sweden	2006 to 2016	Case-control study	Case group: 1631, Control group: 3283	Case group: 1152/479, Control group: 2315/968	Case group: 57 (46,64) Control group: 57 (46,65)	RA: ACR/EULAR 1987 or 2010 criteria
Zaccardelli A (38)	2021	US	The NHS in 1976 and the NHSII in 1989	Cohort study	Case group: 283, Control group: 842	Case group: 283/0, Control group: 842/0	Case group: (51.5 ± 7.6)-(51.5 ± 8.0) Control group: (51.2 ± 7.8)-(51.5 ± 7.9)	RA: The 1987 ACR or 2010 ACR/EULAR criteria
Ford JA (10)	2020	US	June 1, 2014 for NHS and June 1, 2015 for NHSII	Cohort study	Case group: 3573, Control group: 205153	Case group: 3573/0, Control group: 205153/0	Case group: 52.7 Control group: 44.4	RA: The 1987 ACR or 2010 ACR/EULAR criteria
Kronzer VL (34)	2019	US	2009	Case-control study	Case group: 821, Control group: 2455	Case group: 600/221, Control group: 1792/663	(62 ± 14)	RA: The ACR/EULAR 2010 criteria
Mcguire K (35)	2019	Canada	January 1996 to March 2010	Cohort study	Case group: 24625, Control group: 25396	Case group: 16499/8126, Control group: 17015/8381	Case group: (57.2 ± 17.1) Control group: (57.3 ± 17.1)	RA and COPD: ICD-9
Choi IA (30)	2018	Korea, International	NA	Cross-sectional study	Case group: 1050, Control group: 3520	Case group: 872/178, Control group: 3191/329	Case group: (56 ± 12) Control group: (56 ± 13)	NA
Dhital R (31)	2018	US	1 January 2013 to 31 December 2013	Case-control study	Case group: 93750, Control group: 281250	Case group: 346785/121965, Control group: 1040354/365895	Case group: (67.47 ± 0.06) Control group: (67.47 ± 0.08)	RA: ICD-9
Sheen YH (32)	2018	US	January 1, 2002, and December 31, 2007	Case-control study	Case group: 221, Control group: 218	Case group: 156/56, Control group: 154/64	Case group: 52.5 (41.7-65.7) Control group: 54.2 (42.6-66.7)	RA: The 1987 ACR classification criteria
Sparks JA (33)	2018	US	1976 to 2014	Cohort study	Case group: 843, Control group: 8399	Case group: 843/0, Control group: 8399/0	Case group: (59.8 ± 10.0) Control group: (59.8 ± 10.0)	NA
Jo YS (29)	2015	Korea	2010 to 2012	Cross-sectional study	Case group: 744, Control group: 3313	Case group: 0/744, Control group: 0/3313	Case group: (65.02 ± 9.40) Control group: (55.06 ± 10.43)	COPD: A former or current smoker with spirometry-proven airflow limitation (FEV1/FVC<0.70)
Shen TC (28)	2014	China, Taiwan	1998 to 2008	Cohort study	Case group: 28725, Control group: 114900	Case group: 22403/6322, Control group: 89612/25288	Case group: (53.8 ± 13.9) Control group: (53.2 ± 14.3)	RA and COPD: ICD-9
Bieber V (26)	2013	Israel	NA	Cross-sectional study	Case group: 9039, Control group: 15070	Case group: 2001/7038, Control group: 3438/11632	Case group: (60.1 ± 16.9) Control group: (61.1 ± 18.3)	RA: CHS physician, COPD: Taken from the CHS Chronic Diseases Registry
Nannini C (27)	2013	US	January 1, 2006.	Cohort study	Case group: 594, Control group: 596	Case group: 435/159, Control group: 438/158	Case group: (57.8 ± 15.2) Control group: (58.2 ± 15.3)	RA: The 1987 ACR classification criteria
Bergström U (25)	2011	Sweden	Between 1974 and 1992	Case-control study	Case group:290, Control group: 1160	Case group: 139/151, Control group: 556/604	Case group: (47 ± 7.1) Control group: (47 ± 7.1)	RA: The 1987 ACR criteria, COPD: The GOLD criteria

(RA, rheumatoid arthritis; COPD, chronic obstructive pulmonary disease; NHS, Nurses’ Health Study; GOLD, global initiative for obstructive lung disease; ACR, American College of Rheumatology; EULAR, European League Against Rheumatism; CHS, Clalit Health Services; FEV1/FVC, forced expiratory volume in 1s/forced vital capacity; NA, not applicable).

### 3.2 Quality assessment

Using the NOS criteria, cohort and case-control studies achieved an average score of 6.79, with all scoring 5 or higher, indicating moderate to high quality (**Supplementary Table 5**). Meanwhile, cross-sectional studies averaged a score of 4.40 on AHRQ, with two scoring 3, suggesting low to medium quality (**Supplementary Table 6**).

### 3.3 Odds of COPD in participants with RA

**Figure 2:**
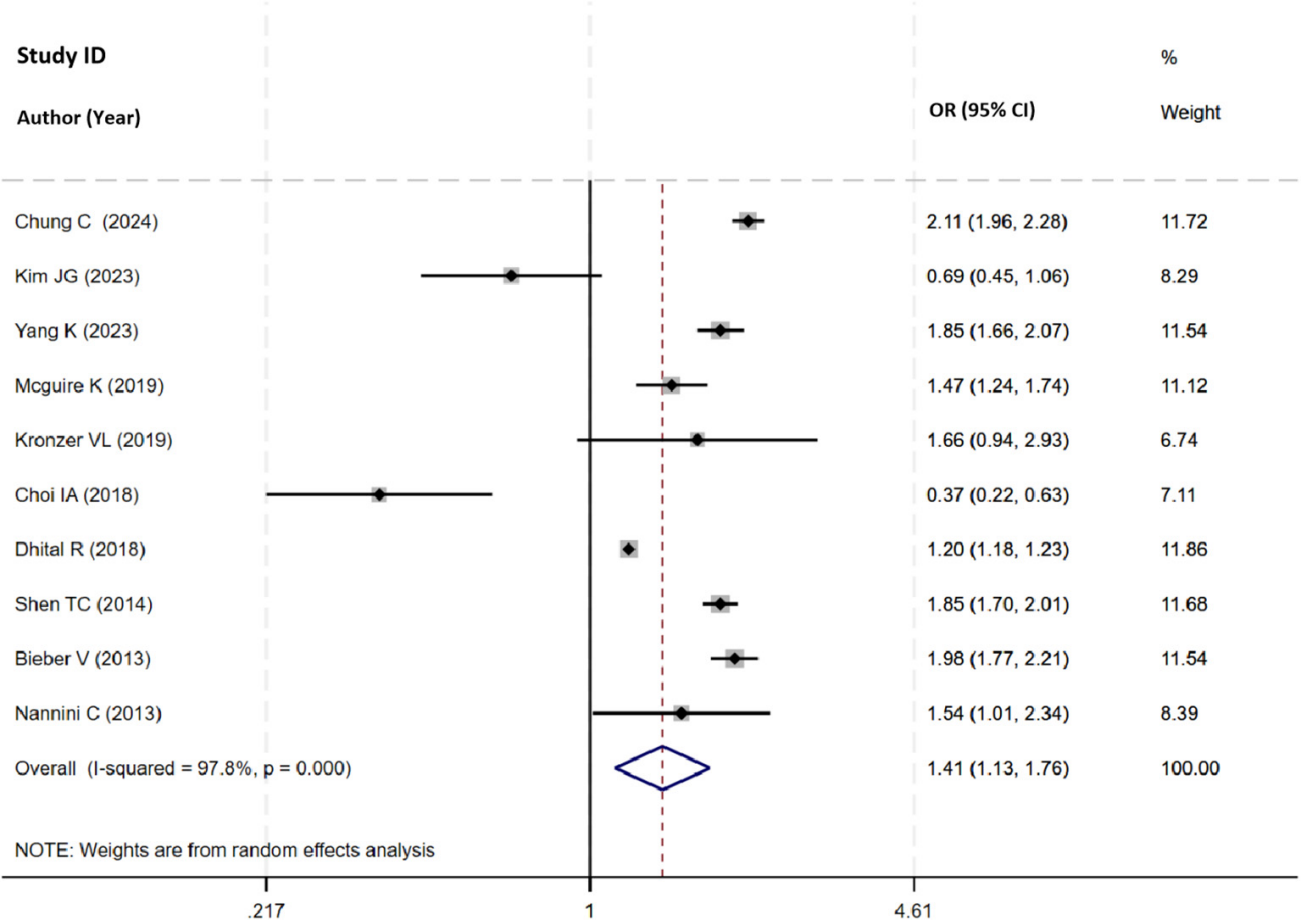
Forest plot showing the odds ratio of COPD in participants with RA.

Thirteen studies investigated the risk of COPD associated with RA, ten studies reported the total OR among these. The pooled analysis confirmed a significant association between RA and increased risk of COPD (OR=1.41, 95% CI 1.13 to 1.76, I^2^ = 97.8%, *P*=0.003, N=10, **Figure 2**), supported by robust sensitivity analysis (**Supplementary Table 7**, **Supplementary Figure 1**).

### 3.4 Odds of RA in participants with COPD

**Figure 3:**
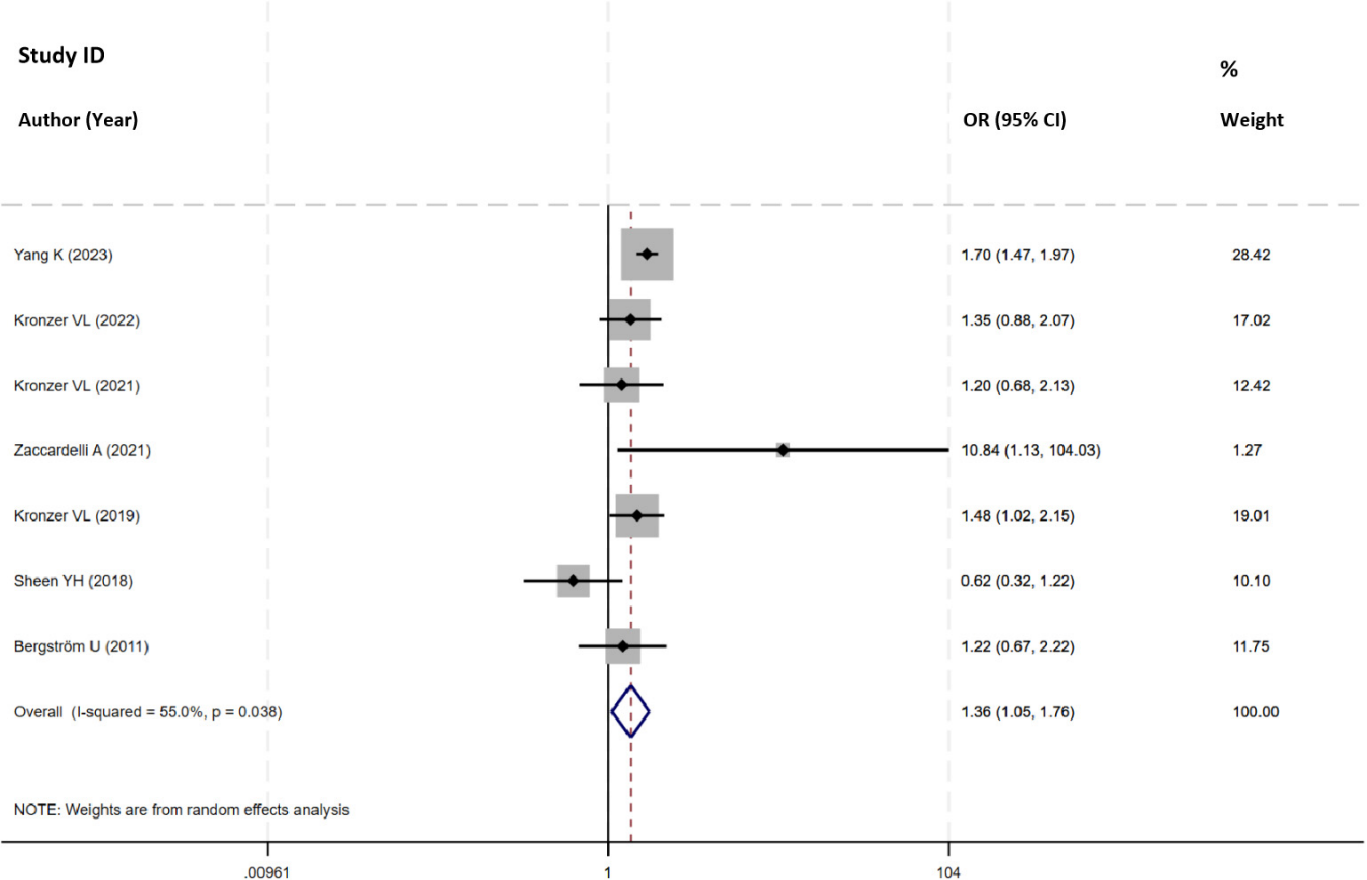
Forest plot showing the odds ratio of RA in participants with COPD.

Nine studies explored the risk of RA linked to COPD, and seven of them reported the total OR. After pooling the data, the analysis revealed a notable association between COPD and an increased risk of RA (OR=1.36, 95%CI 1.05 to 1.76, I^2^ = 55.0%, *P*=0.022, N=7, **Figure 3**). Despite significant heterogeneity attributed to a 2018 study by Sheen YH, its limited sample size didn’t alter the pooled-effect size, reinforcing result robustness (**Supplementary Table 8**, **Supplementary Figure 2**).

### 3.5 Subgroup analysis

In subgroup analysis, the risk of COPD was significantly related to RA in both genders, with females showing a higher risk (OR=1.74, 95% CI 1.59 to 1.90, I^2^ = 0.0%, *P*=0.000) (**Table 2**). Additionally, there was a significant association between seropositive RA and seronegative RA and the risk of COPD, with seropositive RA carried a notably higher COPD risk than seronegative RA (OR=2.09, 95% CI 1.69 to 2.57, I^2^ = 51.4%, *P*=0.000) (**Table 2**). In the subgroup analyses by study design, the meta-analysis of cohort studies and case-control studies showed a significant association between the risk of COPD and RA (OR=1.77, 95%CI 1.60 to 1.97, I^2^ = 71.8%, *P*=0.000; OR=1.43, 95%CI 1.17 to 1.75, I^2^ = 20.0%, *P*=0.028), whereas the meta-analysis of cross-sectional studies suggested a slightly significant link between the risk of COPD and RA (**Table 2**).

**Table 2:** Subgroup analysis for the risk of COPD related to RA.

Subgroups	Included studies	OR (95% CI)	Heterogeneity	
			I^2^ (%)	*P* value
Gender				
Female	5	1.74 (1.59, 1.90)	0.0	0.000
Male	3	1.57 (1.15, 2.14)	69.5	0.004
Serum reactivity				
Seropositive RA	3	2.09 (1.69, 2.57)	51.4	0.000
Seronegative RA	3	1.58 (1.21, 2.06)	50.8	0.001
Study type				
Cohort study	7	1.77 (1.60, 1.97)	71.8	0.000
Cross-sectional study	3	0.81 (0.28, 2.36)	96.4	0.706
Case-control study	2	1.43 (1.17, 1.75)	20.0	0.028

In the subgroup analysis, the risk of RA was significantly linked to COPD in females (OR=1.96, 95%CI 1.37 to 2.81, I^2^ = 0.0%, *P*=0.000), while no significant association was observed in males (OR=0.94, 95% CI 0.56 to 1.59, I^2^ = 0.0%, *P*=0.829) (**Table 3**). There was a significant association between COPD and the risk of both seropositive RA (OR=1.65, 95%CI 1.24 to 2.19, I^2^ = 4.6%, *P*=0.001) and seronegative RA(OR=1.70, 95%CI 1.23 to 2.36, I^2^ = 0.0%, *P*=0.001) (**Table 3**). In the subgroup analyses by study design, the meta-analysis of cohort studies revealed a significant association between the risk of RA and COPD (OR=1.74, 95%CI 1.50 to 1.99, I^2^ = 28.6%, *P*=0.000), whereas meta-analysis of case-control studies and cross-sectional study suggested a slightly significant link between the risk of RA and COPD (**Table 3**).

**Table 3:** Subgroup analysis for the risk of RA associated with COPD.

Subgroups	Included studies	OR (95% CI)	Heterogeneity	
			I^2^ (%)	*P* value
Gender				
Female	2	1.96 (1.37, 2.81)	0.0	0.000
Male	2	0.94 (0.56, 1.59)	0.0	0.829
Serum reactivity				
Seropositive RA	4	1.65 (1.24, 2.19)	4.6	0.001
Seronegative RA	4	1.70 (1.23, 2.36)	0.0	0.001
Study type				
Case-control study	5	1.24 (0.10, 1.52)	21.6	0.051
Cohort study	3	1.74 (1.50, 1.99)	28.6	0.000
Cross-sectional study	1	0.92 (0.40, 2.12)	–	0.845

### 3.6 GRADE quality of evidence

The GRADE level of evidence for the risk of COPD related to RA was assessed as very low. Similarly, the GRADE level was very low for the risk of COPD in males, seropositive RA, cohort studies, and cross-sectional studies, and low for the risk of COPD in females, seronegative RA, and case control study. The GRADE level of evidence was also very low for the risk of RA association with COPD. For the risk of RA in females, males, seropositive/seronegative RA, case-control studies, and cohort studies, the GRADE level of evidence was low. GRADE evidence certainty for the outcomes was summarized in **Supplementary Table 9**.

### 3.7 Publication bias

Funnel plots showed no publication bias for bidirectional risks between COPD and RA, confirmed by Egger’s test (**Supplementary Figures 3**, **4**).

## 4 Discussion

### 4.1 Main findings

Our meta-analysis of 19 studies, encompassing 1,549,181 individuals, elucidated the bidirectional association between COPD and RA. The findings indicated a significant increase in the risk of COPD among individuals with RA compared to those without RA, suggesting that RA may serve as an independent risk factor for COPD. Subgroup analyses by gender, seropositive and seronegative RA, and cohort studies consistently supported this association.

Moreover, our analysis indicated a notable increase in the risk of RA among individuals with COPD, suggesting COPD as an independent contributor to RA development. Subgroup analyses, particularly those focusing on gender, seropositive RA, and seronegative RA, further underscored this association.

Overall, these findings highlight a reciprocal relationship between COPD and RA, suggesting that each condition may predispose individuals to an elevated risk of developing the other. This comprehensive assessment adds valuable insights into the complex interplay between these two chronic inflammatory diseases.

### 4.2 Interpretation of findings

Two earlier reviews, from 2016 and 2019, explored the increased risk of COPD associated with RA (15, 16). The 2019 review included six studies, four of which overlapped with those from 2016 (27, 28, 43, 44), and added two additional articles (33, 35). Additionally, the 2019 review conducted subgroup analyses, further underscored the relationship between RA and heightened COPD risk in North America, Europe, and Asia. Furthermore, the 2019 review also showed a pooled COPD prevalence of 6.2% among RA patients, emphasized by subgroup analyses.

Our meta-analysis, incorporated 13 studies, underscored this bidirectional risks between COPD and RA. Whereas, we eliminated two studies that included in earlier reviews (43, 44), for one study utilized different outcome indicators (43), and another included patients with inflammatory arthritis beyond RA (44). Our study included nine additional studies, providing a comprehensive assessment of the RA-COPD relationship, and also identified nine studies demonstrating a significant association between COPD and RA risk. This underscores the bidirectional nature of their relationship, investigating both the likelihood of COPD in RA participants and the odds of RA in COPD participants. We further found the bidirectional association to be significant in the studies. Furthermore, our article featured subgroup analyses pertaining to gender, serum reactivity, and study type, aspects not previously explored in meta-analyses.

The studies reported RA is more common in female with three- to five-fold higher prevalence of RA in females than males, and the global prevalence ratio of RA is about 1%, with small continuous fluctuations and an apparent growth from south to north, and from countryside to metropolitan areas (1, 45). This may explain the results in the subgroup analysis of gender.

Several potential mechanisms may explain the association between RA and COPD. The genetic susceptibility to RA correlates with increased COPD risk, particularly early-onset (46). Although mendelian randomization studies support this association, causality has not been definitively established (47). Additionally, specific HLA alleles, like shared epitope-positive 04 alleles, are associated with higher airway disease incidence in RA patients, while HLA-DRB11502 shows a negative correlation (48). Further research is required to fully grasp the genetic ties and underlying mechanisms between RA and COPD.

Smoking is a recognized risk factor for both COPD and RA, with a significant impact on RA development, especially in seropositive cases (49). Smoking contributed to the production of RA-specific autoantibodies like ACPA, anti-cyclic citrullinated peptide (anti-CCP) antibodies (50–52). Furthermore, smoking interacts with specific RA genetic risk factors, like the HLA-DRB1 shared epitope (SE), increasing the risk of ACPA-positive RA, particularly, in SE carriers (53, 54). Additionally, smoking contributes to COPD. The harmful chemicals and particles in cigarette smoke trigger abnormal inflammatory responses in the lungs, leading to tissue damage, airway narrowing, and progressive loss of lung function seen in COPD. Overall, smoking not only increases the risk of developing RA by promoting autoimmune processes such as citrullination and anti-CCP antibody production, but also contributes to the development and progression of COPD through its damaging effects on the respiratory system. Avoiding smoking is crucial for reducing the risk of both RA and COPD and improving overall health.

There are shared pathophysiological features between COPD and RA. Elevated levels of rheumatoid factor and anti-CCP antibodies are associated with COPD, indicating a potential overlap in autoimmune processes. Citrullination of autoantigens, a process implicated in RA, lead to the subsequent production of ACPA, which is increased in COPD (13, 55). Interestingly, individuals with ACPA positivity before RA development have a higher risk of COPD, suggesting ACPAs may play a role in COPD pathogenesis (38). Furthermore, the risk of COPD is higher in patients with seropositive RA compared to seronegative RA in this study, indicating a bidirectional association between RA-associated autoimmune processes and COPD inflammatory pathways. Indeed, systemic inflammation in RA can lead to pulmonary dysfunction, including COPD. Immune cells like neutrophils, macrophages, and T-lymphocytes, pivotal in both diseases, contribute to lung tissue damage and inflammation. Moreover, key cytokines such as TNF-α, IL-6, IL-8, IL-17, and IL-32 drive inflammation, tissue destruction, and remodeling in the lungs, aiding COPD progression (56). Autoimmune processes in RA and chronic inflammation in COPD create an environment where cytokines and immune cells interact, damaging both joints and lungs. Understanding these shared pathways is crucial for developing effective therapeutic strategies for patients with both conditions.

The interplay between lung bacterial composition (microbiota), inflammation, and autoimmune processes in RA and COPD highlights their intricate connection. Alterations in the lung microbiome and inflammatory markers occur in both diseases, indicating a significant link between microbial imbalance and systemic inflammation (57, 58). Chronic infection and microbiome irregularities may trigger autoantigen formation, leading to RA-specific markers like ACPA (59). Microbial diversity decline and specific bacterial taxa proliferation correlate with IL-17-mediated immunity, promoting pro-inflammatory cytokine production crucial in both RA and COPD (60). RA patients often endure chronic pulmonary inflammation and recurrent infections, damaging alveolar structures and increasing COPD susceptibility. Addressing lung inflammation and microbiome composition could potentially mitigate COPD progression in RA patients. Understanding these mechanisms better may pave the way for novel therapeutic strategies, emphasizing the importance of preserving lung health in RA to prevent or alleviate COPD.

Moreover, RA may originate at inflamed mucosal sites, including the lungs, potentially linked to upper respiratory viruses (61). Individuals with COPD show elevated inflammatory markers and autoantibodies associated with RA (62). Nonetheless, further research is needed to confirm whether COPD represents a new pathogenic pathway for RA.

### 4.3 Implications and limitations

The strength of our meta-analysis lies in included 19 observational studies, robustly evaluating the RA-COPD association. While two prior meta-analyses examined the risk of COPD in RA and its morbidity, our study offers further retrieved on the bidirectional association relationship between the two conditions. We explore not only the relationship of COPD in individuals with RA but also the odds of RA in those with COPD. Understanding the bidirectional relationship better may benefit for novel therapeutic strategies, emphasizing the importance of preserving lung health to prevent or alleviate COPD, or RA. Understanding the potential link between the two diseases may help clinicians pay closer attention to the progression of the disease and prognosis in time.

The results must be interpreted in light of several limitations. Firstly, these results may be biased due to the unrestricted inclusion of study types, cohort studies, case-control studies, and cross-sectional studies each have their own scopes of application. Secondly, subgroup analyses stratified by gender lacked sufficient statistical power due to limited study availability. Thirdly, some articles relied on ICD codes for defining RA and COPD, which may lack the precision of diagnostic criteria or standardized procedures. Additionally, one study diagnosed COPD based on the CHS Chronic Diseases Registry without elaboration, while two studies omitted mention of diagnostic criteria. Lastly, all studies included in the meta-analysis were conducted in North America, Europe, and Asia, with no representation from African countries. Further large-scale population-based studies are needed to elucidate the significance of the RA-COPD association in African populations.

## 5 Conclusions

In conclusions, this meta-analysis indicates a significant bidirectional association between RA and COPD across the included studies. The risk of COPD was markedly related to RA in both genders, and in both seropositive RA and seronegative RA, meanwhile, there was a significant COPD risk in females RA, and in both seropositive RA and seronegative RA. Early detection of both conditions may be vital for initiating effective treatments and improving long-term outcomes.

## Data Availability

The original contributions presented in the study are included in the article/**Supplementary Material**. Further inquiries can be directed to the corresponding authors.
