# Supplementary material for: Bidirectional association between rheumatoid arthritis and chronic obstructive pulmonary disease: a systematic review and meta-analysis
Source: Front Immunol. 2024 Dec 2;15:1494003. doi: 10.3389/fimmu.2024.1494003 (PMC11647564; doi:10.3389/fimmu.2024.1494003)
Supplement: Supplementary file 1 [file DataSheet1.doc]

**Supplemental Tables**

**Supplementary Table S1-S3:** Details of the Literature Search Strategy

**Supplementary Table 1** PubMed (February 20, 2024)

| Search | Query | Results |
| --- | --- | --- |
| 1 | ((Arthritis, Rheumatoid[MeSH Terms]) OR (Rheumatoid Arthritis[Title/Abstract])) OR (RA[Title/Abstract]) | 208,704 |
| 2 | ((((((((((((Pulmonary Disease, Chronic Obstructive[MeSH Terms]) OR (Chronic Obstructive Lung Disease[Title/Abstract])) OR (Chronic Obstructive Pulmonary Diseases[Title/Abstract])) OR (COAD[Title/Abstract])) OR (COPD[Title/Abstract])) OR (Chronic Obstructive Airway Disease[Title/Abstract])) OR (Chronic Obstructive Pulmonary Disease[Title/Abstract])) OR (Airflow Obstruction, Chronic[Title/Abstract])) OR (Airflow Obstructions, Chronic[Title/Abstract])) OR (Chronic Airflow Obstructions[Title/Abstract])) OR (Chronic Airflow Obstruction[Title/Abstract])) OR (Lung Diseases, Obstructive[Title/Abstract])) OR (Pulmonary Emphysema[Title/Abstract]) | 110,034 |
| 3 | (((Arthritis, Rheumatoid[MeSH Terms]) OR (Rheumatoid Arthritis[Title/Abstract])) OR (RA[Title/Abstract])) AND (((((((((((((Pulmonary Disease, Chronic Obstructive[MeSH Terms]) OR (Chronic Obstructive Lung Disease[Title/Abstract])) OR (Chronic Obstructive Pulmonary Diseases[Title/Abstract])) OR (COAD[Title/Abstract])) OR (COPD[Title/Abstract])) OR (Chronic Obstructive Airway Disease[Title/Abstract])) OR (Chronic Obstructive Pulmonary Disease[Title/Abstract])) OR (Airflow Obstruction, Chronic[Title/Abstract])) OR (Airflow Obstructions, Chronic[Title/Abstract])) OR (Chronic Airflow Obstructions[Title/Abstract])) OR (Chronic Airflow Obstruction[Title/Abstract])) OR (Lung Diseases, Obstructive[Title/Abstract])) OR (Pulmonary Emphysema[Title/Abstract])) | 823 |

**Supplementary Table 2** Cochrane Library (February 20, 2024)

| **Search** | **Query** | **Results** |
| --- | --- | --- |
| #1 | MeSH descriptor:[Arthritis, Rheumatoid] explode all trees | 8,009 |
| #2 | (Rheumatoid Arthritis):ti,ab,kw | 18,868 |
| #3 | RA:ti,ab,kw | 14,485 |
| #4 | #1 OR #2 OR #3 | 23,683 |
| #5 | MeSH descriptor. [Pulmonary Disease, Chronic Obstructive] explode all trees | 8,273 |
| #6 | (Chronic Obstructive Lung Disease):ti,ab,kw | 13,724 |
| #7 | (Chronic Obstructive Pulmonary Diseases):ti,ab,kw | 4,769 |
| #8 | COAD:ti,ab,kw | 99 |
| #9 | COPD:ti,ab,kw | 19,406 |
| #10 | (Chronic Obstructive Airway Disease):ti,ab,kw | 2,101 |
| #11 | (Chronic Obstructive Pulmonary Disease):ti,ab,kw | 17,012 |
| #12 | (Airflow Obstruction, Chronic):ti,ab,kw | 664 |
| #13 | (Airflow Obstructions, Chronic):ti,ab,kw | 2 |
| #14 | (Chronic Airflow Obstructions):ti,ab,kw | 2 |
| #15 | (Chronic Airflow Obstruction):ti,ab,kw | 664 |
| #16 | (Lung Diseases, Obstructive):ti,ab,kw | 5,192 |
| #17 | (Pulmonary Emphysema):ti,ab,kw | 1,150 |
| #18 | #5 OR #6 OR #7 OR #8 OR #9 OR #10 OR #11 OR #12 OR #13 OR #14 OR #15 OR #16 OR #17 | 26,183 |
| #19 | #4 AND #18 | 171 |

**Supplementary Table 3** Embase (February 20, 2024)

| **Search** | **Query** | **Items found** |
| --- | --- | --- |
| #1 | 'Arthritis, Rheumatoid'/exp | 260,987 |
| #2 | 'Rheumatoid Arthritis':ab,ti | 189,960 |
| #3 | 'RA':ab,ti | 160,893 |
| #4 | #1 OR #2 OR #3 | 347,393 |
| #5 | 'Pulmonary Disease, Chronic Obstructive'/exp | 182,750 |
| #6 | 'Chronic Obstructive Lung Disease':ab,ti | 6,805 |
| #7 | 'Chronic Obstructive Pulmonary Diseases':ab,ti | 1,558 |
| #8 | 'COAD':ab,ti | 1,411 |
| #9 | 'COPD':ab,ti | 112,437 |
| #10 | 'Chronic Obstructive Airway Disease':ab,ti | 532 |
| #11 | 'Chronic Obstructive Pulmonary Disease':ab,ti | 89,835 |
| #12 | 'Airflow Obstruction, Chronic':ab,ti | 27 |
| #13 | 'Airflow Obstructions, Chronic':ab,ti | 0 |
| #14 | 'Chronic Airflow Obstructions':ab,ti | 2 |
| #15 | 'Chronic Airflow Obstruction':ab,ti | 701 |
| #16 | 'Lung Diseases, Obstructive':ab,ti | 17 |
| #17 | 'Pulmonary Emphysema ':ab,ti | 5,182 |
| #18 | #5 OR #6 OR #7 OR #8 OR #9 OR #10 OR #11 OR #12 OR #13 OR #14 OR #15 OR #16 OR #17 | 215,127 |
| #19 | #4 AND #18 | 3,952 |
| #20 | 'risk'/exp | 3,180,067 |
| #21 | 'risk':ab,ti | 4,111,308 |
| #22 | #20 OR #21 | 5,045,931 |
| #23 | #19 AND #22 | 1,461 |

**Supplementary Table 4** Details of the excluded studies

|  | Study | Year | Title | Reason for exclued |
| --- | --- | --- | --- | --- |
| 1 | Abumoawad A | 2024 | Trends and outcomes of lytic-based therapies for high-risk pulmonary embolism: A nationwide analysis | The full text does not meet the inclusion criteria |
| 2 | Accorttn A | 2017 | Retrospective analysis to describe associations between tumor necrosis factor alpha inhibitors and COPD-related hospitalizations | The full text does not meet the inclusion criteria |
| 3 | Airoldi C | 2023 | Estimate of the prevalence of subjects with chronic diseases in a province of Northern Italy: a retrospective study based on administrative databases | The full text does not meet the inclusion criteria |
| 4 | Albrecht K | 2014 | Comorbidity in rheumatoid arthriti | The full text does not meet the inclusion criteria |
| 5 | Andreakos E | 2004 | Common and uncommon features of rheumatoid arthritis and chronic obstructive pulmonary disease: clues to a future therapy | The full text does not meet the inclusion criteria |
| 6 | Arkema EV | 2015 | Are patients with rheumatoid arthritis still at an increased risk of tuberculosis and what is the role of biological treatments? | The full text does not meet the inclusion criteria |
| 7 | Aurrecoechea E | 2017 | Gender-associated comorbidities in rheumatoid arthritis and their impact on outcome: data from GENIRA | The full text does not meet the inclusion criteria |
| 8 | Batko B | 2019 | Comorbidity burden and clinical characteristics of patients with difficult-to-control rheumatoid arthritis | The full text does not meet the inclusion criteria |
| 9 | Black RJ | 2023 | Mortality estimates and excess mortality in rheumatoid arthritis | The full text does not meet the inclusion criteria |
| 10 | Brannan HM | 1964 | PULMONARY DISEASE ASSOCIATED WITH RHEUMATOID ARTHRITIS | The full text does not meet the inclusion criteria |
| 11 | Cao Z | 2023 | Causal association of rheumatoid arthritis with obstructive lung disease: Evidence from Mendelian randomization study | The full text does not meet the inclusion criteria |
| 12 | Chang KH | 2015 | A large-scale study indicates increase in the risk of epilepsy in patients with different risk factors, including rheumatoid arthritis | The full text does not meet the inclusion criteria |
| 13 | Chen HH | 2021 | Factors associated with incident severe pulmonary arterial hypertension in systemic autoimmune rheumatic diseases: A nationwide study | The full text does not meet the inclusion criteria |
| 14 | Chen HH | 2020 | Air pollutants and development of interstitial lung disease in patients with connective tissue disease: A population-based case-control study in Taiwan | The full text does not meet the inclusion criteria |
| 15 | Chen YJ | 2021 | Factors associated with risk of major adverse cardiovascular events in patients with rheumatoid arthritis: a nationwide, population-based, case-control study | The full text does not meet the inclusion criteria |
| 16 | Chuang YW | 2016 | Risk of peripheral arterial occlusive disease in patients with rheumatoid arthritis: A nationwide population-based cohort study | The full text does not meet the inclusion criteria |
| 17 | Curtis JR | 2015 | Incidence and complications of interstitial lung disease in users of tocilizumab, rituximab, abatacept and anti-tumor necrosis factor aα agents, a retrospective cohort study | The full text does not meet the inclusion criteria |
| 18 | D’Amico ME | 2021 | Role of comorbidities on therapeutic persistence of biological agents in rheumatoid arthritis: results from the RECord-linkage On Rheumatic Disease study on administrative healthcare databases | The full text does not meet the inclusion criteria |
| 19 | Dong H | 2019 | Interstitial lung abnormalities in patients with early rheumatoid arthritis: A pilot study evaluating prevalence and progression | The full text does not meet the inclusion criteria |
| 20 | Dougados M | 2014 | Prevalence of comorbidities in rheumatoid arthritis and evaluation of their monitoring: Results of an international, cross-sectional study (COMORA) | The full text does not meet the inclusion criteria |
| 21 | Du Rietz E | 2021 | Mapping phenotypic and aetiological associations between ADHD and physical conditions in adulthood in Sweden: a genetically informed register study | The full text does not meet the inclusion criteria |
| 22 | Fan J | 2022 | Multimorbidity patterns and association with mortality in 0.5 million Chinese adults | The full text does not meet the inclusion criteria |
| 23 | Friedlander HM | 2020 | Obstructive lung diseases and risk of rheumatoid arthritis | The full text does not meet the inclusion criteria |
| 24 | Fuller DT | 2021 | Prevalence of Common Comorbidities in Rheumatoid Arthritis in Rural New York Compared With National Data | The full text does not meet the inclusion criteria |
| 25 | Gaik OS | 2022 | Predictors and radiological characteristics of rheumatoid arthritis-associated interstitial lung disease in a multi-ethnic Malaysian cohort | The full text does not meet the inclusion criteria |
| 26 | Gergianaki I | 2019 | Chronic obstructive pulmonary disease and rheumatic diseases: A systematic review on a neglected comorbidity | The full text does not meet the inclusion criteria |
| 27 | Hemminki K | 2011 | Subsequent COPD and lung cancer in patients with autoimmune disease | The full text does not meet the inclusion criteria |
| 28 | Honda Y | 2022 | Multimorbidity patterns and the relation to self-rated health among older Japanese people: A nationwide cross-sectional study | The full text does not meet the inclusion criteria |
| 29 | Huang S | 2019 | Association of rheumatoid arthritis-related autoantibodies with pulmonary function test abnormalities in a rheumatoid arthritis registry | The full text does not meet the inclusion criteria |
| 30 | Hudson M | 2020 | Comparative safety of biologic versus conventional synthetic DMARDs in rheumatoid arthritis with COPD: A real-world population study | The full text does not meet the inclusion criteria |
| 31 | Hyldgaard C | 2018 | Increased mortality among patients with rheumatoid arthritis and COPD: A population-based study | The full text does not meet the inclusion criteria |
| 32 | Hyldgaard C | 2018 | COPD: an overlooked cause of excess mortality in patients with rheumatoid arthritis | The full text does not meet the inclusion criteria |
| 33 | Hyldgaard C | 2022 | Pulmonary manifestations in rheumatoid arthritis | The full text does not meet the inclusion criteria |
| 34 | Innala L | 2016 | Co-morbidity in patients with early rheumatoid arthritis - inflammation matters | The full text does not meet the inclusion criteria |
| 35 | Kang EH | 2020 | Risk of exacerbation of pulmonary comorbidities in patients with rheumatoid arthritis after initiation of abatacept versus TNF inhibitors: A cohort study | The full text does not meet the inclusion criteria |
| 36 | Kim K | 2023 | Factors for progressive pulmonary fibrosis in connective tissue disease-related interstitial lung disease | The full text does not meet the inclusion criteria |
| 37 | Kimbrough BA | 2024 | Multiple morbidities are associated with serious infections in patients with rheumatoid arthritis | The full text does not meet the inclusion criteria |
| 38 | Kuo CF | 2020 | Risk of preventable hospitalization before and after diagnosis among rheumatoid arthritis patients compared to non-rheumatoid arthritis controls | The full text does not meet the inclusion criteria |
| 39 | Kuo CF | 2020 | Risk of preventable hospitalization before and after diagnosis among rheumatoid arthritis patients compared to non-rheumatoid arthritis controls | The full text does not meet the inclusion criteria |
| 40 | Lee H | 2018 | Racial differences in comorbidity profile among patients with chronic obstructive pulmonary disease | The full text does not meet the inclusion criteria |
| 41 | Leone PM | 2020 | Interstitial lung abnormalities a risk factor for rheumatoid arthritis interstitial lung disease progression: What’s new | The full text does not meet the inclusion criteria |
| 42 | Liao TL | 2016 | Risk Factors and Outcomes of Nontuberculous Mycobacterial Disease among Rheumatoid Arthritis Patients: A Case-Control study in a TB Endemic Area | The full text does not meet the inclusion criteria |
| 43 | Lin MC | 2020 | Association of chinese herbal medicines use with development of chronic obstructive pulmonary disease among patients with rheumatoid arthritis: A population-based cohort study | The full text does not meet the inclusion criteria |
| 44 | Lisspers K | 2019 | Gender differences among Swedish COPD patients: results from the ARCTIC, a real-world retrospective cohort study | The full text does not meet the inclusion criteria |
| 45 | Ludvigsson JF | 2012 | A nationwide cohort study of the risk of chronic obstructive pulmonary disease in coeliac disease | The full text does not meet the inclusion criteria |
| 46 | McDermott GC | 2023 | Prevalence and mortality associations of interstitial lung abnormalities in rheumatoid arthritis within a multicentre prospective cohort of smokers | The full text does not meet the inclusion criteria |
| 47 | McFarlane IM | 2019 | Assessment of interstitial lung disease among black rheumatoid arthritis patients | The full text does not meet the inclusion criteria |
| 48 | McLoone P | 2023 | Classification of long-term condition patterns in rheumatoid arthritis and associations with adverse health events: a UK Biobank cohort study | The full text does not meet the inclusion criteria |
| 49 | Ng KH | 2022 | Analysis of risk factors of mortality in rheumatoid arthritis patients with interstitial lung disease: a nationwide, population-based cohort study in Taiwan | The full text does not meet the inclusion criteria |
| 50 | Ni J | 2023 | Arthritis and incident pulmonary diseases in middle-aged and elderly Chinese: a longitudinal population-based study | The full text does not meet the inclusion criteria |
| 51 | Nikiphorou E | 2020 | Prognostic value of comorbidity indices and lung diseases in early rheumatoid arthritis: a UK population-based study | The full text does not meet the inclusion criteria |
| 52 | Norton S | 2013 | A study of baseline prevalence and cumulative incidence of comorbidity and extra-articular manifestations in RA and their impact on outcome | The full text does not meet the inclusion criteria |
| 53 | Norton S | 2013 | A study of baseline prevalence and cumulative incidence of comorbidity and extra-articular manifestations in ra and their impact on outcome | The full text does not meet the inclusion criteria |
| 54 | Panopoulos S | 2021 | Comparable or higher prevalence of comorbidities in antiphospholipid syndrome vs rheumatoid arthritis: a multicenter, case-control study | The full text does not meet the inclusion criteria |
| 55 | Panopoulos S | 2018 | Prevalence of comorbidities in systemic sclerosis versus rheumatoid arthritis: a comparative, multicenter, matched-cohort study | The full text does not meet the inclusion criteria |
| 56 | Pieringer H | 2017 | RABBIT risk score and ICU admission due to infection in patients with rheumatoid arthritis | The full text does not meet the inclusion criteria |
| 57 | Radner H | 2017 | Incidence and Prevalence of Cardiovascular Risk Factors Among Patients With Rheumatoid Arthritis, Psoriasis, or Psoriatic Arthritis | The full text does not meet the inclusion criteria |
| 58 | Shen TC | 2016 | Risk of Chronic Obstructive Pulmonary Disease in Female Adults With Primary Sjögren Syndrome: A Nationwide Population-Based Cohort Study | The full text does not meet the inclusion criteria |
| 59 | Suissa S | 2019 | Comparative safety of abatacept in rheumatoid arthritis with COPD: A real-world population-based observational study | The full text does not meet the inclusion criteria |
| 60 | Thomas E | 2003 | National study of cause-specific mortality in rheumatoid arthritis, juvenile chronic arthritis, and other rheumatic conditions: a 20 year followup study | The full text does not meet the inclusion criteria |
| 61 | Ursum J | 2013 | Increased risk for chronic comorbid disorders in patients with inflammatory arthritis: a population based study | The full text does not meet the inclusion criteria |
| 62 | Watanabe R | 2022 | Prevalence and predictive factors of difficult-to-treat rheumatoid arthritis: the KURAMA cohort | The full text does not meet the inclusion criteria |
| 63 | Wheeler AM | 2022 | Genetic, social, and environmental risk factors in rheumatoid arthritis-associated interstitial lung disease | The full text does not meet the inclusion criteria |
| 64 | Zhao N | 2022 | Fine particulate matter components and interstitial lung disease in rheumatoid arthritis | The full text does not meet the inclusion criteria |
| 65 | Zheng B | 2022 | Association between chronic obstructive pulmonary disease, smoking, and interstitial lung disease onset in rheumatoid arthritis | The full text does not meet the inclusion criteria |

**Supplementary Table 5** Details of the NOS

| Study | Year | Selection | Comparability | Outcome | Overall quality  score |
| --- | --- | --- | --- | --- | --- |
| Chung C | 2024 | ★★★ | ★★ | ★★★ | 8 |
| Yang K | 2023 | ★★★ |  | ★★★ | 6 |
| Kronzer VL | 2022 | ★★★★ | ★★ | ★★ | 8 |
| Kronzer VL | 2021 | ★★ | ★★ | ★★ | 6 |
| Zaccardelli A | 2021 | ★★ | ★★ | ★★ | 6 |
| Ford JA | 2020 | ★★★ |  | ★★ | 5 |
| Kronzer VL | 2019 | ★★★ | ★★ | ★★ | 7 |
| Mcguire K | 2019 | ★★★ | ★★ | ★★ | 7 |
| Dhital R | 2018 | ★★★ | ★★ | ★★ | 7 |
| Sheen YH | 2018 | ★★★ | ★★ | ★★ | 7 |
| Sparks JA | 2018 | ★★★ | ★★ | ★★ | 7 |
| Shen TC | 2014 | ★★★ | ★★ | ★★ | 7 |
| Nannini C | 2013 | ★★★ | ★★ | ★★ | 7 |
| Bergström U | 2011 | ★★★ | ★★ | ★★ | 7 |

**Supplementary Table 6** Details of the AHRQ

| Study | Year | AHRQ Scores |
| --- | --- | --- |
| Kim JG | 2023 | 5 |
| Jung JH | 2021 | 5 |
| Choi IA | 2018 | 3 |
| Jo YS | 2015 | 6 |
| Bieber V | 2013 | 3 |

**Supplementary Table 7 Sensitivity analyses for the risk of COPD associated with RA**

|  |  | **OR** | **95% CI** | **I^2^/%** | ***P* value** |
| --- | --- | --- | --- | --- | --- |
|  | **Total** | 1.41 | 1.12-1.76 | 97.8 | 0.003 |
|  | **Excluded study** |  |  |  |  |
| 1 | Chung C 2024 | 1.34 | 1.08-1.68 | 96.7 | 0.009 |
| 2 | Kim JG 2023 | 1.50 | 1.19-1.90 | 98.0 | 0.001 |
| 3 | Yang K 2023 | 1.36 | 1.06-1.73 | 97.8 | 0.015 |
| 4 | Mcguire K 2019 | 1.40 | 1.09-1.78 | 98.9 | 0.007 |
| 5 | Kronzer VL 2019 | 1.39 | 1.10-1.76 | 98.0 | 0.005 |
| 6 | Choi IA 2018 | 1.56 | 1.24-1.96 | 97.9 | 0.000 |
| 7 | Dhital R 2018 | 1.53 | 1.30-1.80 | 89.4 | 0.000 |
| 8 | Shen TC 2014 | 1.35 | 1.05-1.74 | 97.6 | 0.018 |
| 9 | Bieber V 2013 | 1.35 | 1.06-1.71 | 97.7 | 0.015 |
| 10 | Nannini C 2013 | 1.40 | 1.10-1.77 | 98.0 | 0.006 |

**Supplementary Table 8 Sensitivity analyses for the risk of RA associated with COPD**

|  |  | **OR** | **95% CI** | **I^2^/%** | ***P* value** |
| --- | --- | --- | --- | --- | --- |
|  | **Total** | 1.36 | 1.04-1.76 | 55.0 | 0.022 |
|  | **Excluded study** |  |  |  |  |
| 1 | Yang K 2023 | 1.27 | 1.02-1.58 | 41.8 | 0.032 |
| 2 | Kronzer VL 2022 | 1.34 | 0.98-1.84 | 61.2 | 0.070 |
| 3 | Kronzer VL 2021 | 1.37 | 1.02-1.84 | 60.1 | 0.034 |
| 4 | Zaccardelli A 2021 | 1.34 | 1.05-1.70 | 52.3 | 0.018 |
| 5 | Kronzer VL 2019 | 1.31 | 0.93-1.83 | 62.3 | 0.117 |
| 6 | Sheen YH 2018 | 1.60 | 1.41-1.81 | 15.9 | 0.000 |
| 7 | Bergström U 2011 | 1.37 | 1.02-1.83 | 60.6 | 0.036 |

**Supplementary Table 9** GRADE certainty of evidence

| **Outcomes** | **Exposure** | **Study of findings** | | **Quality assessment** | | | | | **Certainty of**  **evidence** |
| --- | --- | --- | --- | --- | --- | --- | --- | --- | --- |
|  |  | No. studies | OR (95%CI) | Study design^*^ | Inconsistency^†^ | Indirectness | Imprecision | Other consideration |  |
| COPD | RA | 13 | 1.41  (1.13, 1.76) | serious | serious | not serious | not serious | not serious | Very Low |
| COPD | Female | 5 | 1.74  (1.59, 1.90) | serious | not serious | not serious | not serious | not serious | Low |
| COPD | Male | 3 | 1.57  (1.15, 2.14) | serious | serious | not serious | not serious | not serious | Very Low |
| COPD | Seropositive RA | 3 | 2.09  (1.69, 2.57) | serious | serious | not serious | not serious | not serious | Very Low |
| COPD | Seronegative RA | 3 | 1.58  (1.21, 2.06) | serious | not serious | not serious | not serious | not serious | Low |
| COPD | Cohort study | 7 | 1.77  (1.60, 1.97) | serious | serious | not serious | not serious | not serious | Very Low |
| COPD | Cross-sectional study | 3 | 0.81  (0.28, 2.36) | serious | serious | not serious | not serious | not serious | Very Low |
| COPD | Case-control study | 2 | 1.43  (1.17, 1.75) | serious | not serious | not serious | not serious | not serious | Low |
| RA | COPD | 9 | 1.36  (1.05, 1.76) | serious | serious | not serious | not serious | not serious | Very Low |
| RA | Female | 2 | 1.96  (1.37, 2.81) | serious | not serious | not serious | not serious | not serious | Low |
| RA | Male | 2 | 0.94  (0.56, 1.59) | serious | not serious | not serious | not serious | not serious | Low |
| RA | Seropositive RA | 4 | 1.65  (1.24, 2.19) | serious | not serious | not serious | not serious | not serious | Low |
| RA | Seronegative RA | 4 | 1.70  (1.23, 2.36) | serious | not serious | not serious | not serious | not serious | Low |
| RA | Case-control study | 5 | 1.24  (0.10, 1.52) | serious | not serious | not serious | not serious | not serious | Low |
| RA | Cohort study | 3 | 1.74  (1.50, 1.99) | serious | not serious | not serious | not serious | not serious | Low |

(COPD: chronic obstructive pulmonary disease, RA: rheumatoid arthritis, ^*^Downgraded by one level if >25% of participants in this comparison were from studies at high risk of bias. ^†^Downgraded by one level if heterogeneity (I^2^)>50%.)
