# Supplementary material for: Bidirectional association between rheumatoid arthritis and chronic obstructive pulmonary disease: a systematic review and meta-analysis
Source: Front Immunol. 2024 Dec 2;15:1494003. doi: 10.3389/fimmu.2024.1494003 (PMC11647564; doi:10.3389/fimmu.2024.1494003)
Supplement: Supplementary file 2 [file DataSheet2.doc]

**Supplemental** **Figures**

**Supplementary Figure 1** Sensitivity analysis of the risk of COPD associated with RA

**Supplementary Figure 2** Sensitivity analysis of the risk of RA associated with COPD

**Supplementary Figure 3** Publication bias of the risk of COPD associated with RA

**Supplementary Figure 4** Publication bias of the risk of RA associated with COPD
